# Influence of Anatomical Pelvic Tilt and Anatomical Sacral Slope on Pelvic Orientation and Lumbar Lordosis Distribution in Subjects with High Pelvic Incidence

**DOI:** 10.3390/jcm15187309

**Published:** 2026-09-20

**Authors:** Norio Imai, Daisuke Homma, Makoto Shirono, Yuki Endo, Yuki Hirano, Yoji Horigome, Hiroyuki Kawashima

**Affiliations:** 1 Division of Comprehensive Musculoskeletal Medicine, Niigata University Graduate School of Medicine, Dentistry and Health Sciences, Niigata 951-8510, Japan; 2 Division of Orthopedic Surgery, Department of Regenerative and Transplant Medicine, Niigata University Graduate School of Medicine, Dentistry and Health Sciences, Niigata 951-8510, Japan; 3 Department of Rehabilitation, Niigata Bandai Hospital, Niigata 950-0909, Japan; 4 Department of Orthopedic Surgery, Niigata Prefectural Shibata Hospital, Niigata 957-0054, Japan

**Keywords:** pelvic incidence, anterior pelvic plane, sacral slope, pelvic tilt, lower arc lordosis

## Abstract

**Background/Objectives:** Pelvic incidence (PI) is a key anatomical parameter that influences sagittal spinal alignment and hip–spine mechanics. Anatomically, PI is composed of anatomical pelvic tilt (aPT) and anatomical sacral slope (aSS). However, whether variation in these anatomical components is associated with lumbar lordosis distribution in subjects with high PI remains unclear. This study aimed to investigate the association between aPT and distal lumbar lordosis distribution in subjects with high PI. **Methods:** This retrospective cross-sectional study included 320 subjects, of whom 68 had high PI (PI ≥ 60°). Sacral slope (SS), pelvic tilt (PT), anterior pelvic plane angle (APPA), PI, aPT, aSS, thoracic kyphosis (TK), lumbar lordosis (LL), lower arc lordosis (L4–S1 lordosis), and the L4–S1/LL ratio were measured using standing lateral radiographs. In subjects with high PI, associations of aPT as a continuous variable with lower arc lordosis and the L4–S1/LL ratio were evaluated using correlation and multiple linear regression analyses adjusted for age and body mass index (BMI). **Results:** Sixty-eight subjects (21.3%) met the definition of high PI. Greater aPT was modestly associated with greater lower arc lordosis in unadjusted analysis (Pearson r = 0.258, *p* = 0.034), whereas the association with the L4–S1/LL ratio did not reach statistical significance (r = 0.219, *p* = 0.072). After adjustment for age and BMI, the association between aPT and lower arc lordosis was attenuated and did not reach statistical significance (standardized β = 0.217, *p* = 0.063). The adjusted association between aPT and the L4–S1/LL ratio was also not significant (standardized β = 0.191, *p* = 0.113). **Conclusions:** Among subjects with high PI, greater aPT showed a modest positive association with distal lumbar lordosis in unadjusted analysis; however, this association was attenuated after adjustment for age and BMI. These findings suggest that age may partly contribute to variation in distal lumbar lordosis among individuals with high PI and that the anatomical composition of PI should be interpreted with consideration of potential confounding factors.

## 1. Introduction

Pelvic incidence (PI) is a fundamental anatomical parameter that plays a central role in determining sagittal spinal alignment. Previous studies have demonstrated that greater PI is generally associated with greater sacral slope (SS) and lumbar lordosis (LL), contributing to the maintenance of sagittal balance [1,2,3,4,5,6,7,8]. Furthermore, high PI has been associated with hyperlordotic lumbar alignment, increased facet joint loading, and a higher prevalence of degenerative spondylolisthesis [4,9,10,11,12]. Consequently, PI has become one of the most important parameters in the evaluation of sagittal spinal balance and spinal disorders.

In addition to its influence on spinal alignment, PI has also been implicated in hip morphology and hip biomechanics. Previous studies have demonstrated that spinopelvic alignment differs in patients with hip osteoarthritis and developmental dysplasia of the hip, suggesting a close interaction between pelvic morphology and hip function. Furthermore, PI has been shown to influence functional hip anatomy, acetabular orientation, and hip–spine mechanics. Therefore, PI is increasingly recognized as a key parameter not only in spinal disorders but also in hip pathology and total hip arthroplasty [11,13,14,15].

The clinical significance of PI extends beyond spinal pathology. In recent years, increasing attention has been directed toward the relationship between the spine, pelvis, and hip joint, commonly referred to as the hip–spine relationship. Several studies have demonstrated that pelvic morphology and spinopelvic alignment influence hip function, acetabular orientation, and postoperative outcomes following total hip arthroplasty (THA) [13,16,17,18]. In particular, pelvic tilt and spinopelvic mobility have been shown to alter functional acetabular anteversion and inclination, thereby affecting impingement risk, prosthetic stability, and dislocation after THA [13,16,17,18]. Therefore, a detailed understanding of pelvic morphology and pelvic orientation has become increasingly important in contemporary hip surgery.

From an anatomical perspective, PI is geometrically composed of pelvic tilt (PT) and sacral slope (SS) [1] as a functional parameter, and anatomical pelvic tilt (aPT) and anatomical sacral slope (aSS), according to the relationship PI = PT + SS [1], as well as aPT + aSS [1,10]. Unlike functional PT and SS, which vary according to posture, aPT and aSS are anatomical parameters that reflect intrinsic pelvic morphology. We previously reported that patients with developmental dysplasia of the hip exhibited greater PI and greater aPT than healthy individuals, while a strong correlation existed between PI and aSS [1]. Subsequent studies further demonstrated that aSS is closely associated with PI and may serve as a useful surrogate parameter for estimating PI [19,20,21,22].

However, despite sharing a similar PI value, individuals may exhibit substantially different combinations of aPT and aSS. In other words, one individual may have a relatively large aPT and small aSS, whereas another may have a relatively small aPT and large aSS despite having the same PI. This observation raises the possibility that different anatomical mechanisms may contribute to the development of a similarly high PI. Whether these differences influence spinal alignment or pelvic orientation remains largely unknown.

We have previously demonstrated that anterior pelvic plane angle (APPA), a parameter representing pelvic orientation, correlates more strongly with aPT than with PI itself [23]. In addition, we previously demonstrated that patients with developmental dysplasia of the hip exhibit greater PI and aPT values than healthy individuals, suggesting that variations in the anatomical components of PI may influence pelvic morphology and orientation [14,23]. These findings suggest that aPT may influence pelvic posture independently of global spinal alignment. However, few studies have investigated the influence of the relative contribution of aPT and aSS in subjects with similarly high PI values. Moreover, although high PI is generally associated with greater lumbar lordosis, it remains unclear whether differences in aPT and aSS contribute to variations in lumbar lordosis distribution, particularly within the distal lumbar spine [24,25].

Clarifying these issues may provide new insights into spinopelvic morphology and the hip–spine relationship. Furthermore, understanding the anatomical factors that influence pelvic orientation may have important implications for preoperative planning in THA, where functional acetabular orientation is strongly affected by pelvic posture.

Therefore, the purpose of this study was to investigate whether differences in the anatomical components of PI are associated with pelvic orientation and sagittal spinal alignment in subjects with high PI. We hypothesized that subjects with aSS-dominant morphology would demonstrate greater functional SS and greater lumbar lordosis, particularly greater lower arc lordosis, than subjects with aPT-dominant morphology. In addition, to avoid loss of information from dichotomization, we examined the associations of aPT as a continuous anatomical parameter with distal lumbar lordosis distribution.

## 2. Materials and Methods

### 2.1. Participants

This retrospective study included patients who visited the osteoporosis outpatient clinic at Niigata Prefectural Shibata Hospital between April 2015 and December 2021. A total of 384 patients who were referred to our clinic for the diagnosis and treatment of osteoporosis were initially screened. Of these, 6 patients with a history of spinal surgery and 58 patients with radiographically identified vertebral fractures were excluded. Consequently, 320 subjects were included in the final analysis.

### 2.2. Radiographic Measurements

**Figure 1:**
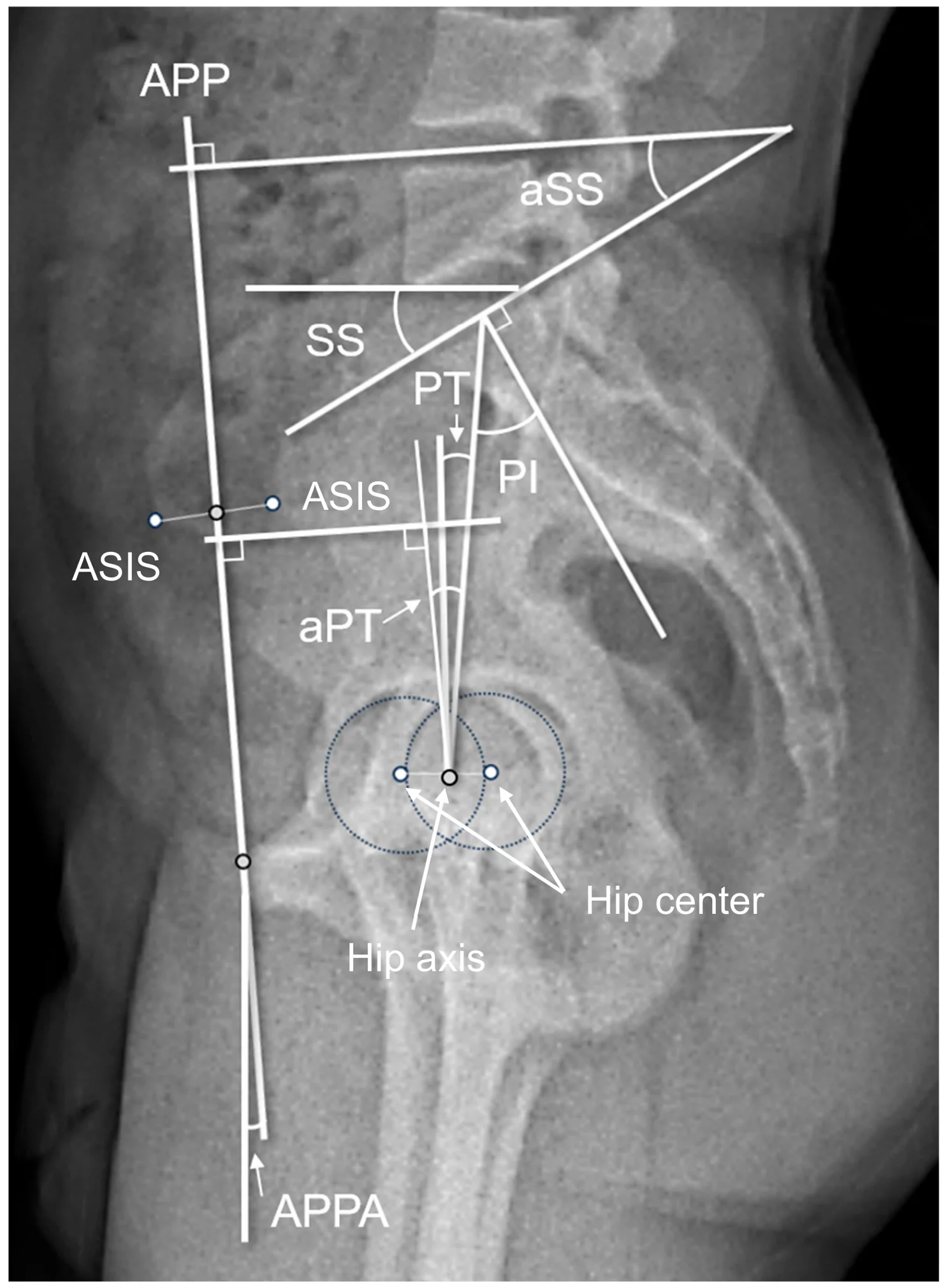
Pelvic sagittal parameters. Illustration of the pelvic sagittal parameters measured on standing lateral radiographs. Pelvic incidence (PI), pelvic tilt (PT), sacral slope (SS), anatomical pelvic tilt (aPT), anatomical sacral slope (aSS), anterior pelvic plane angle (APPA), and the anterior pelvic plane (APP) are shown. PI is defined as the sum of PT and SS, whereas anatomically PI corresponds to the sum of aPT and aSS (PI = PT + SS = aPT + aSS).

Lateral standing radiographs of the entire spine including the pelvis were obtained for all subjects. All radiographic measurements were performed using a picture archiving and communication system (PACS; SYNAPSE, version 5, FUJIFILM Corporation, Tokyo, Japan). Functional pelvic parameters, including sacral slope (SS), pelvic tilt (PT), and anterior pelvic plane angle (APPA), were measured (Figure 1). APPA was defined as the angle between the anterior pelvic plane (APP), which is formed by both anterior superior iliac spines and the most anterior point of the pubic symphysis, and the vertical axis.

Anatomical pelvic parameters were also evaluated. Pelvic incidence (PI) was measured according to the conventional definition. Anatomical sacral slope (aSS) and anatomical pelvic tilt (aPT) were defined as the angles formed between the vertical axis of the APP and SS and PT, respectively (Figure 1) [14,19,20,21].

**Figure 2:**
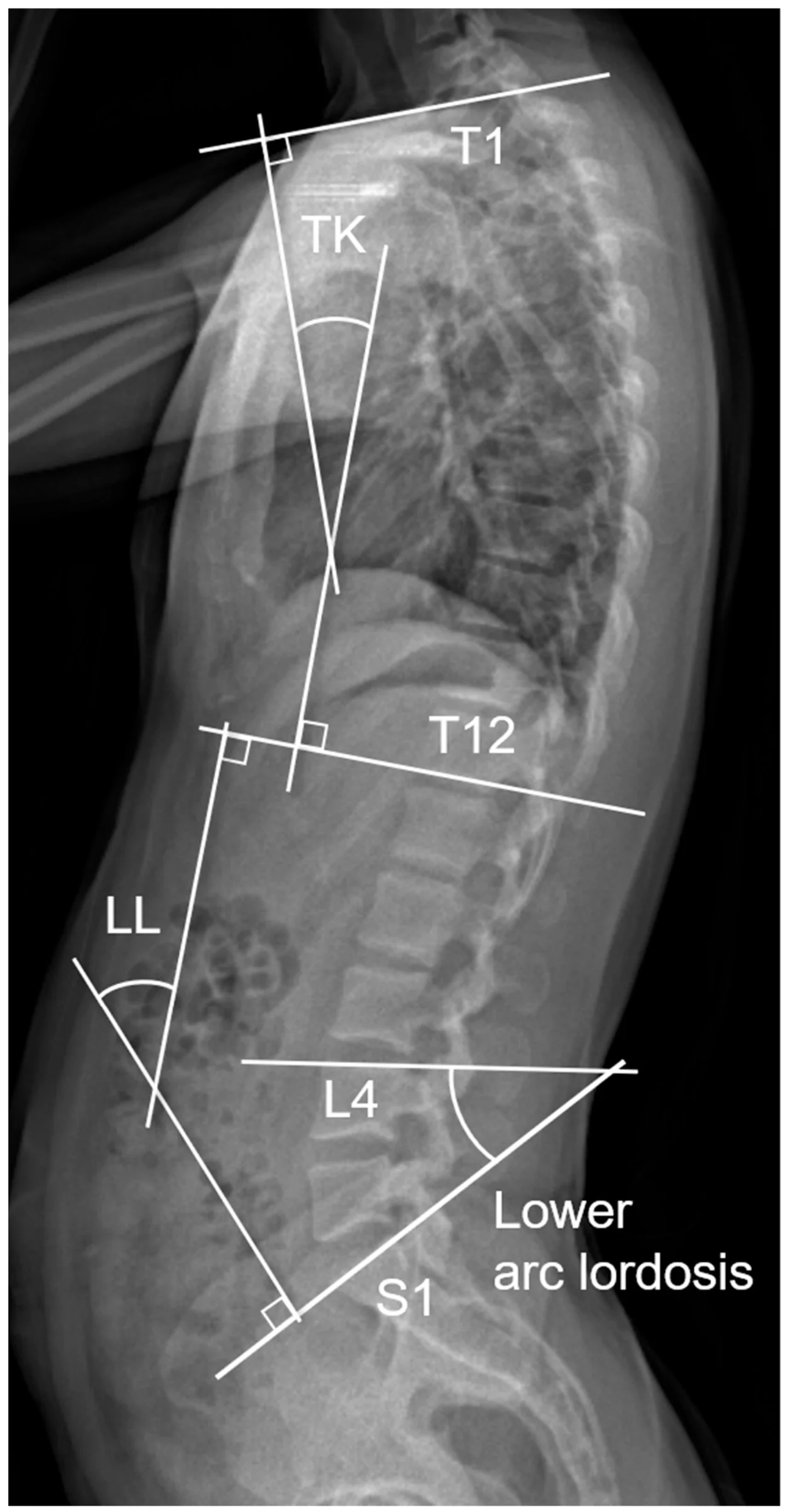
Spinal sagittal parameters. Illustration of the spinal sagittal parameters measured on standing lateral radiographs. Thoracic kyphosis (TK) was measured between the superior endplate of T1 and the inferior endplate of T12. Lumbar lordosis (LL) was measured between the superior endplate of L1 and the superior endplate of S1. Lower arc lordosis was defined as the angle between the superior endplate of L4 and the superior endplate of S1.

Spinal sagittal alignment parameters included thoracic kyphosis (TK) and lumbar lordosis (LL). To evaluate distal lumbar lordosis, lower arc lordosis was defined as the angle between the superior endplate of L4 and the superior endplate of S1 (Figure 2) [4,5,10,26].

### 2.3. Definition of High Pelvic Incidence and Evaluation of Anatomical Pelvic Parameters

Previous studies have reported mean PI values ranging from approximately 50° to 54° in asymptomatic adult populations. Therefore, a PI value of 60° corresponds approximately to one standard deviation above the population mean and is generally considered representative of a high-PI morphology. Furthermore, this threshold is consistent with the concept of Roussouly type 4 alignment, which is characterized by high PI, high SS, and hyperlordotic lumbar alignment. Accordingly, high PI was defined as PI ≥ 60° in the present study [1,4,27,28,29].

Subjects with PI ≥ 60° were extracted. For descriptive comparison, these subjects were classified according to the median value of the aPT/aSS ratio in the study cohort; subjects with an aPT/aSS ratio greater than or equal to the median value were assigned to the aPT-dominant group, whereas those with a ratio below the median value were assigned to the aSS-dominant group. Because aPT and aSS are geometrically related through PI (PI = aPT + aSS), the primary inferential analyses treated aPT as a continuous variable rather than relying on dichotomization of the aPT/aSS ratio.

### 2.4. Statistical Analysis

Statistical analyses were performed using SPSS version 28 (IBM Corp., Armonk, NY, USA). For descriptive purposes, characteristics of the aPT-dominant and aSS-dominant groups were summarized as means and standard deviations, with between-group mean differences and 95% confidence intervals. No inferential hypothesis testing was performed for these descriptive group comparisons. The primary inferential analyses treated aPT as a continuous variable. In subjects with high PI, Pearson’s correlation coefficients were calculated to evaluate the associations of aPT with lower arc lordosis and the L4–S1/LL ratio. Multiple linear regression analyses were subsequently performed with lower arc lordosis and the L4–S1/LL ratio as dependent variables and aPT as the primary independent variable, adjusting for age and BMI. Age was included as a covariate because previous work demonstrated age-related changes in aPT and spinopelvic alignment [16]. Standardized regression coefficients (β) and *p*-values were reported. Multicollinearity was assessed using variance inflation factors (VIFs). A two-sided *p*-value < 0.05 was considered statistically significant for the primary inferential analyses. Measurement reliability was evaluated using intraclass correlation coefficients (ICCs). For intraobserver reliability, all parameters were measured twice by the same observer with an interval of at least two weeks. For interobserver reliability, two independent observers measured all parameters once, and the resulting values were compared. ICCs were reported with 95% confidence intervals. Standard errors of measurement (SEMs) were calculated as SD × √(1 − ICC), using the standard deviation of the corresponding measurement values. Mean absolute measurement differences were also calculated for each parameter.

### 2.5. Ethical Considerations

This study was approved by the Ethics Committee of Niigata University (Approval No. 2026-0090). Because this was a retrospective cross-sectional study without any intervention, an opt-out approach was adopted, and the requirement for written informed consent was waived by the ethics committee.

### 2.6. AI Statement

During the preparation of this manuscript, the authors used ChatGPT (GPT-5.6 Sol, OpenAI) for assistance with English-language editing, refinement of the manuscript text, and preparation of responses to reviewers. The authors have reviewed and edited the output and take full responsibility for the content of this publication.

## 3. Results

Of the 320 study subjects, 68 (21.3%) met the definition of high PI (PI ≥ 60°). Based on the median aPT/aSS ratio (0.240), 33 subjects were classified into the aPT-dominant group and 35 into the aSS-dominant group.

For descriptive purposes, the mean age was 57.8 years in the aPT-dominant group and 63.1 years in the aSS-dominant group, and the mean BMI was 23.4 and 22.6 kg/m^2^, respectively (Table 1). No inferential hypothesis testing was performed for these descriptive group comparisons.

Descriptively, PI, SS, LL, and TK were similar between the aPT-dominant and aSS-dominant groups (PI, 65.7° vs. 65.4°; SS, 53.2° vs. 53.7°; LL, 60.8° vs. 57.8°; TK, 38.8° vs. 35.2°) (Table 1).

APPA differed descriptively between the aPT-dominant and aSS-dominant groups (7.0° vs. −3.1°). However, because APPA is geometrically related to the anatomical and functional pelvic parameters (APPA = SS − aSS = aPT − PT), this between-group difference was not interpreted as an independent empirical finding.

In the descriptive group comparison, lower arc lordosis was greater in the aPT-dominant group (35.9° vs. 29.8°), and the L4–S1/LL ratio was also greater (0.598 vs. 0.524). In the primary continuous analysis among subjects with high PI, aPT showed a modest positive correlation with lower arc lordosis (Pearson r = 0.258, *p* = 0.034), whereas the correlation between aPT and the L4–S1/LL ratio did not reach statistical significance (r = 0.219, *p* = 0.072). Age was negatively correlated with lower arc lordosis (r = −0.325, *p* = 0.007), whereas the correlation between age and aPT was not significant (r = −0.131, *p* = 0.288). In multiple linear regression analysis adjusted for age and BMI, the association between aPT and lower arc lordosis was attenuated and did not reach statistical significance (standardized β = 0.217, *p* = 0.063). Age remained independently associated with lower arc lordosis (standardized β = −0.292, *p* = 0.013). The adjusted association between aPT and the L4–S1/LL ratio was also not statistically significant (standardized β = 0.191, *p* = 0.113). No evidence of multicollinearity was observed (all VIFs ≤ 1.018).

Measurement reliability for all radiographic parameters is presented in Table 2. Intraobserver ICCs ranged from 0.712 to 0.896, and interobserver ICCs ranged from 0.683 to 0.866. For lower arc lordosis, the intraobserver ICC was 0.864 (95% CI, 0.728–1.000), with an SEM of 0.96°, whereas the interobserver ICC was 0.732 (95% CI, 0.508–0.956), with an SEM of 1.97°. The corresponding mean absolute measurement differences were 2.8 ± 2.2° and 3.4 ± 3.6°, respectively.

## 4. Discussion

The principal finding of the present study was that, among subjects with high PI, aPT showed a modest positive association with lower arc lordosis when analyzed as a continuous variable. However, this association was attenuated after adjustment for age and BMI and did not reach statistical significance. Similarly, no independent association was observed between aPT and the L4–S1/LL ratio after adjustment. These findings indicate that the anatomical composition of PI alone may not independently determine distal lumbar lordosis distribution and that age should be considered when interpreting morphological differences among individuals with high PI.

SS, LL, and TK were comparable between the descriptive groups. PI is a major anatomical determinant of sagittal spinal alignment, and greater PI is generally associated with greater lumbar lordosis [1,2,3,4,5,6,7,8,25]. In the present high-PI cohort, however, the similar global sagittal parameters between groups do not support the original hypothesis that aSS-dominant morphology is associated with greater SS or LL. Accordingly, these descriptive group comparisons were not interpreted as evidence of distinct biomechanical phenotypes.

APPA differed descriptively between the groups; however, because APPA is geometrically related to aPT, aSS, PT, and SS, this difference should not be interpreted as an independent association. Accordingly, the revised inferential analyses focused on the relationship between continuous aPT and lumbar lordosis distribution.

Previous studies have demonstrated that pelvic orientation is clinically relevant to functional acetabular orientation and hip–spine mechanics [13,17,18]. However, the present study did not evaluate dynamic spinopelvic motion, acetabular orientation, impingement, instability, or other clinical outcomes. Therefore, the present findings should be interpreted primarily as morphological observations rather than evidence of specific biomechanical or clinical consequences.

When aPT was treated as a continuous variable, greater aPT was modestly correlated with greater lower arc lordosis in the unadjusted analysis, whereas its association with the L4–S1/LL ratio was not statistically significant. After adjustment for age and BMI, neither association reached statistical significance. These results suggest that the apparent differences in distal lumbar lordosis observed in the descriptive group comparison may be partly influenced by age rather than by the anatomical composition of PI alone. This interpretation is consistent with the importance of considering age-related changes in spinopelvic alignment [6,7,8,20,27,28,29,30].

The original hypothesis was not supported. We hypothesized that subjects with aSS-dominant morphology would demonstrate greater SS and lumbar lordosis, particularly greater lower arc lordosis. However, neither SS nor total LL differed meaningfully between the groups, and lower arc lordosis was greater in the opposite direction in the descriptive group comparison. Furthermore, when aPT was analyzed continuously, its association with lower arc lordosis was attenuated after adjustment for age and BMI. These findings emphasize the importance of considering potential confounding factors and avoiding interpretation based solely on dichotomized anatomical phenotypes.

Age appeared to be an important factor in the present analysis. Age was negatively associated with lower arc lordosis, whereas its correlation with aPT was not statistically significant. Thus, the descriptive aPT-dominant and aSS-dominant groups should not simply be regarded as age strata; nevertheless, adjustment for age attenuated the association between aPT and lower arc lordosis. This finding suggests that age-related variation in lumbar alignment should be considered when evaluating the relationship between pelvic morphology and distal lumbar lordosis [6,7,8,20,24,25,27,28,29,30]. Accordingly, all primary regression analyses were adjusted for age.

Taken together, the continuous analyses provide more limited evidence than the initial dichotomized group comparison. Although aPT may be related to distal lumbar lordosis in subjects with high PI, the present data do not establish distinct biomechanical phenotypes or specific compensatory mechanisms. Further studies with larger samples, dynamic imaging, and clinical outcomes are required to determine the functional significance of variation in the anatomical components of PI.

Previous studies have reported mean PI values of approximately 50–54° in asymptomatic adults [1,6,7,8,27,28,29,30], including Japanese participants [28]. In the present study, 21.3% of subjects met the definition of high PI (PI ≥ 60°), which is consistent with the expected proportion of individuals with PI values approximately one standard deviation above the population mean. Therefore, the selected high-PI cohort was considered representative of a high-PI morphology rather than an extreme subgroup.

This study has several limitations. First, this was a retrospective cross-sectional study conducted at a single institution; therefore, causal relationships cannot be established. Although a modest association between aPT and lower arc lordosis was observed in unadjusted analysis, this association was attenuated after adjustment for age and BMI. The relatively small number of subjects with high PI may also have limited statistical power to detect modest independent associations. Future studies with larger samples and longitudinal designs are required to clarify these relationships.

Second, the study population consisted of patients who attended an osteoporosis outpatient clinic rather than a community-based population. Therefore, selection bias may have been present, and caution is warranted when generalizing the present findings to the general population. In addition, because the high-PI cohort consisted predominantly of women, the present findings may not be generalizable to men.

Third, only static standing radiographs were evaluated. Dynamic spinopelvic motion and changes between standing and sitting positions were not assessed; therefore, the present results cannot be extrapolated to spinopelvic mobility or functional acetabular orientation.

Fourth, lumbar lordosis distribution was evaluated using lower arc lordosis and the L4–S1/LL ratio. More detailed assessments, including lordosis apex location and segmental lordosis, were not performed and may provide additional information regarding the relationship between aPT and lumbar alignment.

Finally, the present study did not evaluate clinically relevant outcomes such as spinal degeneration, hip function, functional acetabular orientation, impingement, or dislocation after total hip arthroplasty. Therefore, no conclusions regarding such clinical or biomechanical consequences can be drawn from the present data. Future studies should investigate whether variation in aPT within high-PI morphology is associated with dynamic spinopelvic behavior or clinical outcomes.

Despite these limitations, the present study demonstrates the importance of analyzing the anatomical components of PI as continuous variables and of accounting for potential confounding factors such as age when evaluating distal lumbar lordosis distribution in subjects with high PI.

Further studies incorporating larger cohorts, dynamic spinopelvic evaluations, and clinical outcomes are required to determine whether variation in the anatomical components of PI has functional significance for hip–spine mechanics.

## 5. Conclusions

Among subjects with high PI, aPT showed a modest positive association with lower arc lordosis in unadjusted analysis; however, this association was attenuated and did not reach statistical significance after adjustment for age and BMI. No significant adjusted association was observed between aPT and the L4–S1/LL ratio.

These findings suggest that age may contribute to variation in distal lumbar lordosis among individuals with high PI and that the anatomical components of PI should be interpreted with consideration of potential confounding factors. Further studies are required to determine the clinical and biomechanical significance of these anatomical variations.

## Figures and Tables

**Table 1:** Descriptive characteristics of the aPT-dominant and aSS-dominant groups.

Variable	aPT Dominant (*n* = 33) *	aSS Dominant (*n* = 35) *	Mean Difference (95% CI)
Age (years)	57.8 ± 11.0	63.1 ± 11.9	−5.3 (−10.9 to 0.2)
Sex (Male/Female)	3/30	0/35	
BMI (kg/m^2^)	23.4 ± 4.4	22.6 ± 2.6	0.7 (−1.1 to 2.5)
LL (°)	60.8 ± 8.4	57.8 ± 10.8	3.0 (−1.7 to 7.7)
Lower arc lordosis (°)	35.9 ± 7.5	29.8 ± 8.5	6.1 (2.2 to 10.0)
L4–S1/LL ratio	0.598 ± 0.148	0.524 ± 0.149	0.074 (0.002 to 0.146)
TK (°)	38.8 ± 10.5	35.2 ± 13.6	3.6 (−2.3 to 9.5)
PT (°)	12.6 ± 8.2	11.6 ± 7.3	0.9 (−2.8 to 4.7)
SS (°)	53.2 ± 7.3	53.7 ± 8.3	−0.6 (−4.3 to 3.2)
APPA (°)	7.0 ± 8.0	−3.1 ± 8.0	10.1 (6.2 to 13.9)
PI (°)	65.7 ± 5.0	65.4 ± 4.8	0.4 (−2.0 to 2.7)
aSS (°)	46.2 ± 8.5	56.8 ± 6.0	−10.6 (−14.2 to −7.0)
aPT (°)	19.5 ± 7.4	8.6 ± 3.3	11.0 (8.1 to 13.8)
aPT/aSS	0.480 ± 0.378	0.156 ± 0.067	0.324 (0.188 to 0.459)

* Values are presented as mean ± standard deviation. Between-group comparisons are descriptive; no inferential hypothesis testing was performed. Mean differences are presented with 95% confidence intervals.

**Table 2:** Intraobserver and interobserver reliability of radiographic measurements.

Parameter	Intraobserver Mean Absolute Difference ± SD	Intraobserver ICC (95% CI)	Intraobserver SEM	Interobserver Mean Absolute Difference ± SD	Interobserver ICC (95% CI)	Interobserver SEM
LL (°)	2.9 ± 2.3	0.840 (0.680–1.000)	1.08	3.6 ± 3.9	0.703 (0.471–0.935)	2.34
Lower arc lordosis (°)	2.8 ± 2.2	0.864 (0.728–1.000)	0.96	3.4 ± 3.6	0.732 (0.508–0.956)	1.97
TK (°)	3.2 ± 2.8	0.727 (0.512–0.942)	1.67	3.6 ± 3.6	0.705 (0.474–0.936)	2.12
APPA (°)	2.7 ± 2.3	0.861 (0.722–1.000)	0.97	3.7 ± 3.3	0.726 (0.501–0.951)	1.94
SS (°)	2.5 ± 2.2	0.896 (0.792–1.000)	0.84	3.8 ± 3.2	0.866 (0.732–1.000)	1.32
PT (°)	2.8 ± 2.8	0.821 (0.642–1.000)	1.02	3.9 ± 3.2	0.684 (0.440–0.928)	1.97
PI (°)	3.8 ± 2.6	0.712 (0.499–0.925)	1.66	4.4 ± 3.8	0.683 (0.438–0.928)	2.36
aSS (°)	2.8 ± 2.4	0.884 (0.768–1.000)	0.92	3.5 ± 2.4	0.856 (0.712–1.000)	1.06
aPT (°)	3.1 ± 3.0	0.722 (0.510–0.934)	1.74	3.9 ± 3.4	0.688 (0.444–0.932)	2.07

Abbreviations: LL, lumbar lordosis; TK, thoracic kyphosis; APPA, anterior pelvic plane angle; SS, sacral slope; PT, pelvic tilt; PI, pelvic incidence; aSS, anatomical sacral slope; aPT, anatomical pelvic tilt; ICC, intraclass correlation coefficient; CI, confidence interval; SEM, standard error of measurement; SD, standard deviation. SEM was calculated as SD × √(1 − ICC), using the standard deviation of the corresponding measurement values.

## Data Availability

The data presented in this study are available on reasonable request from the corresponding author. The data are not publicly available due to ethical restrictions and the protection of participant privacy.
